# An improved YOLOv10-based framework for knee MRI lesion detection with enhanced small object recognition and low contrast feature extraction

**DOI:** 10.3389/frai.2025.1675834

**Published:** 2026-01-20

**Authors:** Hongwei Yang, Wenqu Song, Tiankai Jiang, Chuanhao Wang, Luping Zhang, Zhian Cai, Yuhan Sun, Qing Zhao, Yuyu Sun

**Affiliations:** 1 Department of Orthopaedics, Affiliated Nantong Hospital 3 of Nantong University, Nantong, China; 2 Department of Orthopaedics, Nantong Third People's Hospital, Nantong, China; 3 School of Medicine, Nantong University, Nantong, Jiangsu, China

**Keywords:** knee MRI, lesion detection, low contrast feature extraction, small object detection, YOLOv10

## Abstract

**Rationale and objectives:**

To address the challenges in detecting anterior cruciate ligament (ACL) lesions in knee MRI examinations, including difficulties in identifying tiny lesions, insufficient extraction of low-contrast features, and poor modeling of irregular lesion morphologies, and to provide a precise and efficient auxiliary diagnostic tool for clinical practice.

**Materials and methods:**

An enhanced framework based on YOLOv10 is constructed. The backbone network is optimized using the C2f-SimAM module to enhance multi-scale feature extraction and spatial attention; an Adaptive Spatial Fusion (ASF) module is introduced in the neck to better fuse multi-scale spatial features; and a novel hybrid loss function combining Focal-EIoU and KPT Loss is employed. To ensure rigorous statistical evaluation, we utilized a five-fold cross-validation strategy on a dataset of 917 cases.

**Results:**

Evaluation on the KneeMRI dataset demonstrates that the proposed model achieves statistically significant improvements over standard YOLOv10, Faster R-CNN, and Transformer-based detectors (RT-DETR). Specifically, mAP@0.5 is increased by 1.3% (*p* < 0.05) compared to the standard YOLOv10, and mAP@0.5:0.95 is improved by 2.5%. Qualitative analysis further confirms the model's ability to reduce false negatives in small, low-contrast tears.

**Conclusion:**

This framework effectively connects general object detection models with the specific requirements of medical imaging, providing a precise and efficient solution for diagnosing ACL injuries in routine clinical workflows.

## 1 Introduction

Magnetic resonance imaging (MRI) has emerged as an indispensable modality in modern musculoskeletal diagnostics, offering high-resolution and non-invasive visualization of soft tissue structures. Among various orthopedic applications, the assessment of anterior cruciate ligament (ACL) lesions occupies a pivotal role due to the high incidence of ACL injuries in athletic and general populations. Early and accurate detection of ACL lesions is crucial, as delayed or missed diagnoses can lead to progressive joint degeneration, secondary injuries, and suboptimal treatment outcomes (Chavez et al., 2025; Griffin et al., 2000). Recent regenerative medicine has seen MSCs and their EVs as a new cartilage repair direction for ACL post-injury reconstruction (Yang et al., 2025). Precise preoperative imaging diagnosis is a prerequisite for realizing individualized treatment of traditional surgery or emerging regenerative therapies.

However, automatic detection of ACL lesions in MRI scans presents formidable challenges. Clinically, ACL tears often manifest as subtle signal hyperintensities within the femoral intercondylar notch, which can be easily obscured by artifacts or mimic mucoid degeneration (Liu F. et al., 2018). Secondly, the low contrast between damaged ligaments and surrounding soft tissues further complicates lesion delineation (Sun et al., 2021). Thirdly, MRI data are susceptible to artifacts, noise, and patient-specific anatomical variability (He et al., 2020).

In recent years, deep learning has revolutionized medical image analysis (Litjens et al., 2017). The field of artificial intelligence in medical imaging continues to experience rapid growth, addressing a wide array of diagnostic and prognostic challenges across various modalities (Li et al., 2024). Within this domain, the You Only Look Once (YOLO) family has garnered significant attention due to its balance of accuracy and computational efficiency (Redmon et al., 2016). However, applying generic detectors to medical imaging requires adaptation. Medical images differ fundamentally from natural images in texture homogeneity and object scale (Roth et al., 2018). Conventional architectures often struggle with the “micro-fractures” and subtle fiber disruptions typical of early ACL injury.

To address these limitations, we propose an enhanced YOLOv10-based framework specifically optimized for knee MRI. Unlike previous studies that apply off-the-shelf models, our contributions focus on medical-specific adaptations: (1) Integrating C2f-SimAM modules to capture low-contrast features typical of edema; (2) Introducing an Adaptive Spatial Fusion (ASF) module to handle the irregular morphology of torn ligaments; and (3) Implementing a hybrid Focal-EIoU + KPT loss for precise boundary regression. We validate our approach using rigorous five-fold cross-validation against state-of-the-art models, including Transformers and two-stage detectors.

## 2 Related work

### 2.1 Object detection in medical imaging

Convolutional neural networks (CNNs) have made remarkable progress in object detection. While segmentation models like U-Net are prevalent in medical imaging for pixel-level tasks, object detection frameworks are often preferred for rapid screening and localization of specific pathologies where bounding boxes suffice for clinical decision support. YOLO has been widely adopted; for instance, Zhang et al. (2021) proposed a YOLO-based method for liver tumor detection. More recently, an enhanced YOLOv8 framework, SCFAST-YOLO, was developed for accurate classification of distal radius fractures, showcasing the versatility of YOLO in diverse medical contexts (Wang Y. et al., 2025). However, standard YOLO models often prioritize speed over the fine-grained precision required for orthopedic diagnosis. Recent advancements in low-light image recognition (Gen et al., 2025) and advanced signal processing (Aldanma et al., 2024) suggest that attention mechanisms and enhanced feature fusion are critical for improving performance in challenging visual environments, a concept we adapt here for MRI analysis.

### 2.2 Knee MRI lesion detection

Knee MRI lesion detection, particularly for ACL injuries, presents unique challenges. Existing literature has largely focused on classification (tear vs. no tear) using 2D or 3D CNNs. However, detection (localization) provides more interpretability. Comparative studies often lack rigor, failing to compare against non-YOLO architectures like Faster R-CNN or emerging Transformer-based models (e.g., DETR, Zhu et al., 2021). In this work, we aim to address these gaps by enhancing YOLOv10 and providing a comprehensive comparison against SOTA architectures to benchmark its clinical utility.

## 3 Materials and methods

### 3.1 Dataset demographics and preparation

We utilized the KneeMRI dataset consisting of 917 cases. We specifically selected sagittal plane images for training and evaluation. This decision is based on clinical consensus that the ACL runs obliquely through the knee and is best visualized and graded in the sagittal plane, which offers the highest diagnostic value for ligamentous integrity.

The demographic characteristics of the patient cohort are detailed in Table 1. The dataset includes a balanced representation of gender and laterality.

**Table 1:** Demographic characteristics of the study population.

**Characteristic**	**Value (*N* = 917)**
Age (years), Mean ± SD	32.4 ± 11.2
**Gender**	
Male	568 (61.9%)
Female	349 (38.1%)
**Laterality**	
Right knee	486 (53.0%)
Left knee	431 (47.0%)

To ensure rigorous evaluation, we employed stratified random sampling to divide the dataset into Training, Validation, and Test sets (70:15:15), ensuring the class distribution remained consistent across subsets. The detailed class distribution is presented in Table 2.

**Table 2:** Class distribution across Training, Validation, and Test sets.

**Class**	**Total cases**	**Training (70%)**	**Validation (15%)**	**Test (15%)**
Healthy	550	385	82	83
Partial tear	150	105	23	22
Complete tear	217	152	32	33
Total	917	642	137	138

Data augmentation was performed online during training to improve generalization. Techniques included random horizontal flip (probability 0.5), random rotation (±10°), and mosaic augmentation. The final sample sizes for each epoch varied dynamically due to the mosaic technique, but the base dataset remained fixed as described.

### 3.2 Overall framework architecture

The proposed architecture builds upon the YOLOv10 foundation, incorporating a multi-stage processing pipeline designed to extract and fuse highly discriminative features suitable for complex medical imaging tasks. First, the input MRI slices are passed through an optimized backbone network integrated with C2f-SimAM modules, which effectively capture both local fine-grained features and global semantic representations. The extracted multi-scale feature maps are then passed through an improved neck structure, which typically involves path aggregation mechanisms similar to PANet (Liu S. et al., 2018), incorporating the Adaptive Spatial Fusion module to achieve dynamic multi-scale feature integration. Finally, the head outputs refined bounding box predictions and keypoint estimations under the supervision of a hybrid loss function, which guides the network to achieve highly accurate localization and boundary delineation. This architecture is carefully balanced to ensure both high accuracy and computational efficiency.

**Figure 1:**
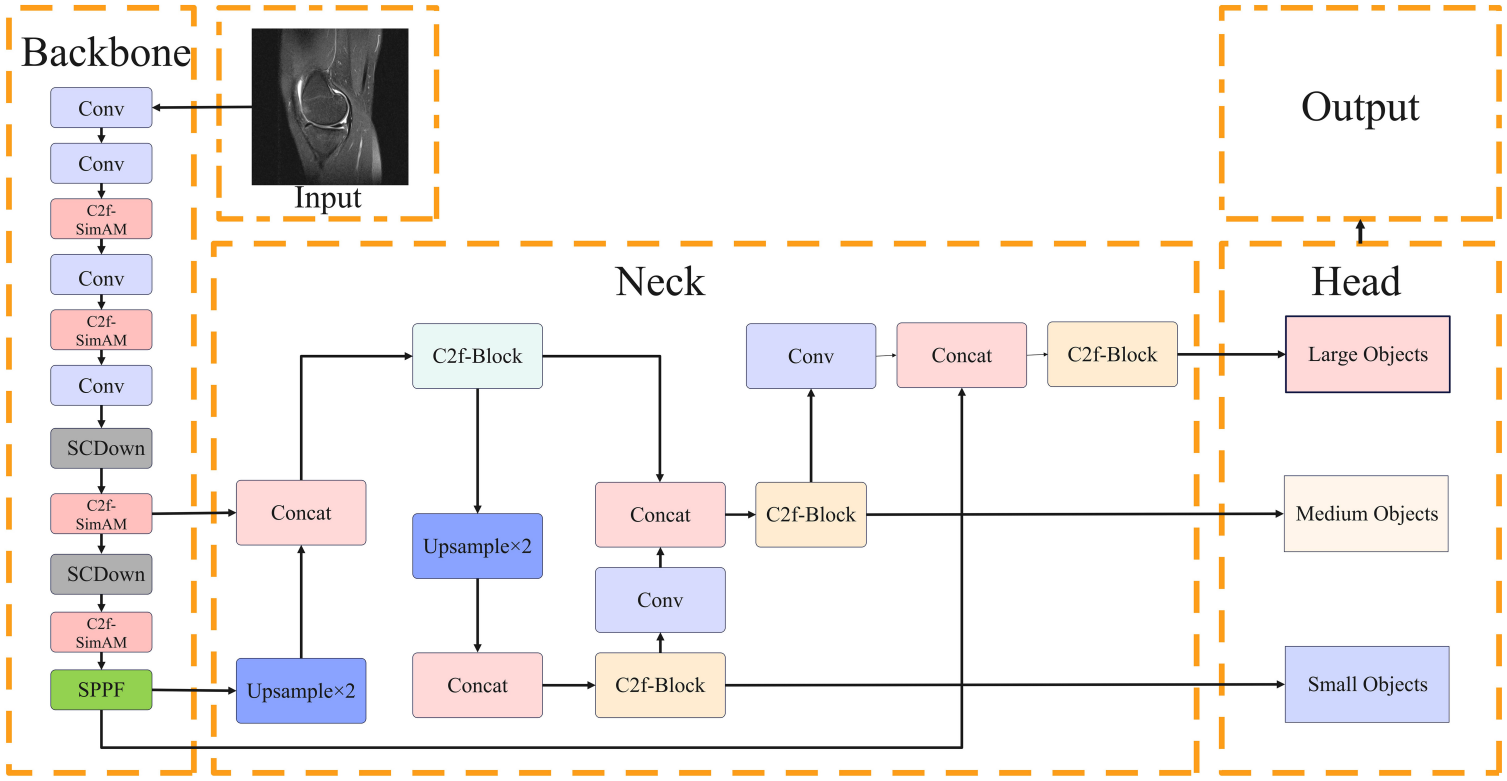
Proposed YOLOv10-based architecture with C2f-SimAM, ASF, and hybrid loss integration for knee MRI lesion detection.

For clarity, we present a visual overview of our enhanced YOLOv10 architecture in Figure 1. We explicitly define key abbreviations here: ACL (Anterior Cruciate Ligament), ASF (Adaptive Spatial Fusion), and SimAM (Simple Attention Module).

### 3.3 Backbone optimization with C2f-SimAM

The original YOLOv10 backbone utilizes Cross Stage Partial (CSP) modules to achieve a trade-off between feature extraction quality and computational cost. However, due to the distinct nature of knee MRI images, where lesions often present as small, low-contrast, and irregularly shaped structures embedded within complex tissue backgrounds, the standard CSP module may not sufficiently capture relevant lesion features. To enhance sensitivity and robustness, we introduce the C2f-SimAM module as a replacement for selected CSP blocks.

Attention mechanisms have significantly advanced deep learning performance. A pioneering example, the Squeeze-and-Excitation (SE) network, introduced a channel-wise attention module to adaptively recalibrate feature responses (Hu et al., 2017). Building on such impactful strategies, our C2f-SimAM module integrates advanced feature processing with effective attention.

The Cross-Stage Fusion (C2f) module partitions the feature map into dual pathways: one branch preserves high-resolution fine-grained spatial features, while the other extracts deeper semantic representations through additional convolutional layers. The outputs of both branches are then concatenated to form a comprehensive feature map:


$$\begin{array}{l} F_{\text{out}}=\text{Concat}\left(F_{1},F_{2}\right) \end{array}$$ (1)

This design enables the network to simultaneously model fine local textures and broader contextual information, which is particularly crucial for detecting minute ACL tears and differentiating them from surrounding normal tissues.

Following C2f processing, the Simple Attention Module (SimAM) is employed to further enhance spatial attention without introducing additional trainable parameters, thereby maintaining computational efficiency. For each neuron *x*_*i*_, SimAM calculates an energy function based on its deviation from the local mean μ and variance σ^2^:


$$\begin{array}{l} E_{i}={\left(x_{i}-\mu \right)}^{2}+\sigma^{2} \end{array}$$ (2)

The corresponding attention weight is computed as:


$$\begin{array}{l} w_{i}=\frac{1}{E_{i}+\epsilon } \end{array}$$ (3)

where ϵ is a stabilizing constant. This mechanism allows the model to emphasize neurons that carry salient lesion-specific information, improving detection accuracy for low-contrast structures typically observed in MRI scans. Such attention-driven feature refinement, including modules integrating both channel and spatial awareness (Woo et al., 2018), is crucial for robust medical image analysis.

### 3.4 Adaptive Spatial Fusion in the neck

**Figure 2:**
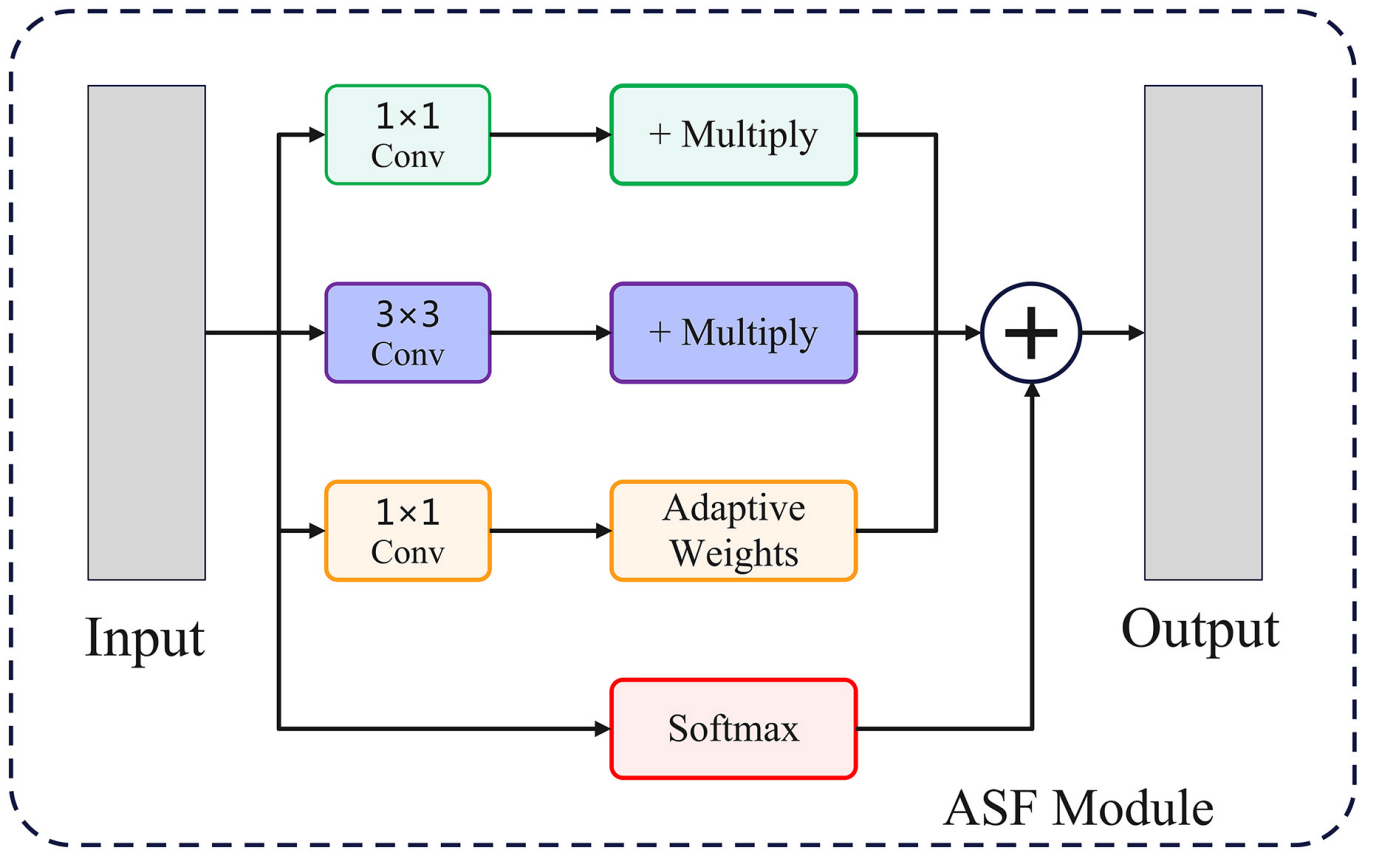
This figure shows the structural flowchart of the ASF module, which is mainly used to achieve adaptive fusion of multi-scale features to enhance the model's ability to detect complex targets.

Feature fusion plays a pivotal role in accurately localizing lesions of varying sizes and morphologies. Traditional neck designs employ fixed fusion rules that may not adapt well to the heterogeneous nature of lesion appearances. To address this, we propose an Adaptive Spatial Fusion (ASF) module, which dynamically assigns attention weights to features at different resolutions. The structure of the module is shown in the Figure 2.

The fused feature representation is calculated as:


$$\begin{array}{l} F_{fused}=\overset{N}{\underset{i=1}{\sum }}\alpha_{i}F_{i} \end{array}$$ (4)

where *F*_*i*_ denotes the feature map from scale *i*, and α_*i*_ are learnable weights normalized to ensure $\overset{N}{\underset{i=1}{\sum }}\alpha_{i}=1$. Through adaptive weighting, the ASF module selectively emphasizes scales that contain the most relevant spatial information for each lesion instance. This capability is particularly beneficial for handling lesions exhibiting diverse shapes, such as elongated ACL tears or fragmented partial injuries.

Additionally, ASF mitigates information loss typically caused by repeated downsampling in conventional neck structures, thereby preserving both fine-grained and global lesion descriptors.

### 3.5 Loss function optimization: focal-EIoU with KPT loss

Accurate lesion detection requires both precise bounding box localization and fine-grained delineation of lesion boundaries. To jointly optimize these objectives, we design a hybrid loss function that integrates Focal-EIoU loss and KeyPoint (KPT) loss.

For bounding box regression, we utilize Focal-EIoU loss, which extends the traditional IoU loss by applying a modulating factor to focus learning on challenging examples (Lin et al., 2017b) and by incorporating aspect ratio penalties:


$$\begin{array}{l} L_{\text{Focal-EIoU}}={\left(1-IoU\right)}^{\gamma }\cdot \left(1-\frac{IoU-v}{1-v}\right) \end{array}$$ (5)

Here, γ controls the focusing strength, and *v* penalizes aspect ratio inconsistencies, which helps stabilize training for lesions of diverse shapes.

Simultaneously, the KPT loss supervises anatomical keypoint predictions to refine lesion boundary alignment. This loss is computed as:


$$\begin{array}{l} L_{\text{KPT}}=\overset{K}{\underset{j=1}{\sum }}|p_{j}-{\hat{p}}_{j}{|}^{2} \end{array}$$ (6)

where *p*_*j*_ and ${\hat{p}}_{j}$ represent the predicted and ground truth keypoint coordinates, respectively.

The combined loss function is formulated as:


$$\begin{array}{l} L_{\text{total}}=\lambda_{1}L_{\text{Focal-EIoU}}+\lambda_{2}L_{\text{KPT}} \end{array}$$ (7)

where λ_1_ and λ_2_ control the relative contributions of each loss component. This joint formulation ensures balanced optimization between coarse localization and fine boundary accuracy, which is highly desirable for medical diagnosis.

### 3.6 Implementation details and hyperparameter selection

The model was implemented in PyTorch. Hyperparameters were optimized using a genetic algorithm (GA) evolution strategy on the validation set for the first 50 epochs. The final parameters were: initial learning rate 0.01 (SGD optimizer), momentum 0.937, and weight decay 0.0005. Transfer learning was employed by initializing the backbone with COCO-pretrained weights to accelerate convergence.

## 4 Results

### 4.1 Experimental setup and statistical analysis

To ensure the robustness of our results, we implemented a five-fold cross-validation scheme. All reported metrics represent the mean ± standard deviation across the five fold. Statistical significance was evaluated using the paired *t*-test, with a *p*-value < 0.05 considered statistically significant. Confidence intervals (95% CI) were calculated for the primary metric (mAP).

### 4.2 Evaluation metrics

To evaluate detection performance, we adopt widely accepted object detection metrics: precision (P), recall (R), mean average precision (mAP@0.5), mAP@0.5:0.95 (multi-scale average), inference time per image (ms), and floating-point operations (FLOPs). These metrics allow us to comprehensively assess both detection accuracy and computational efficiency.

### 4.3 Attention mechanism visualization

**Figure 3:**
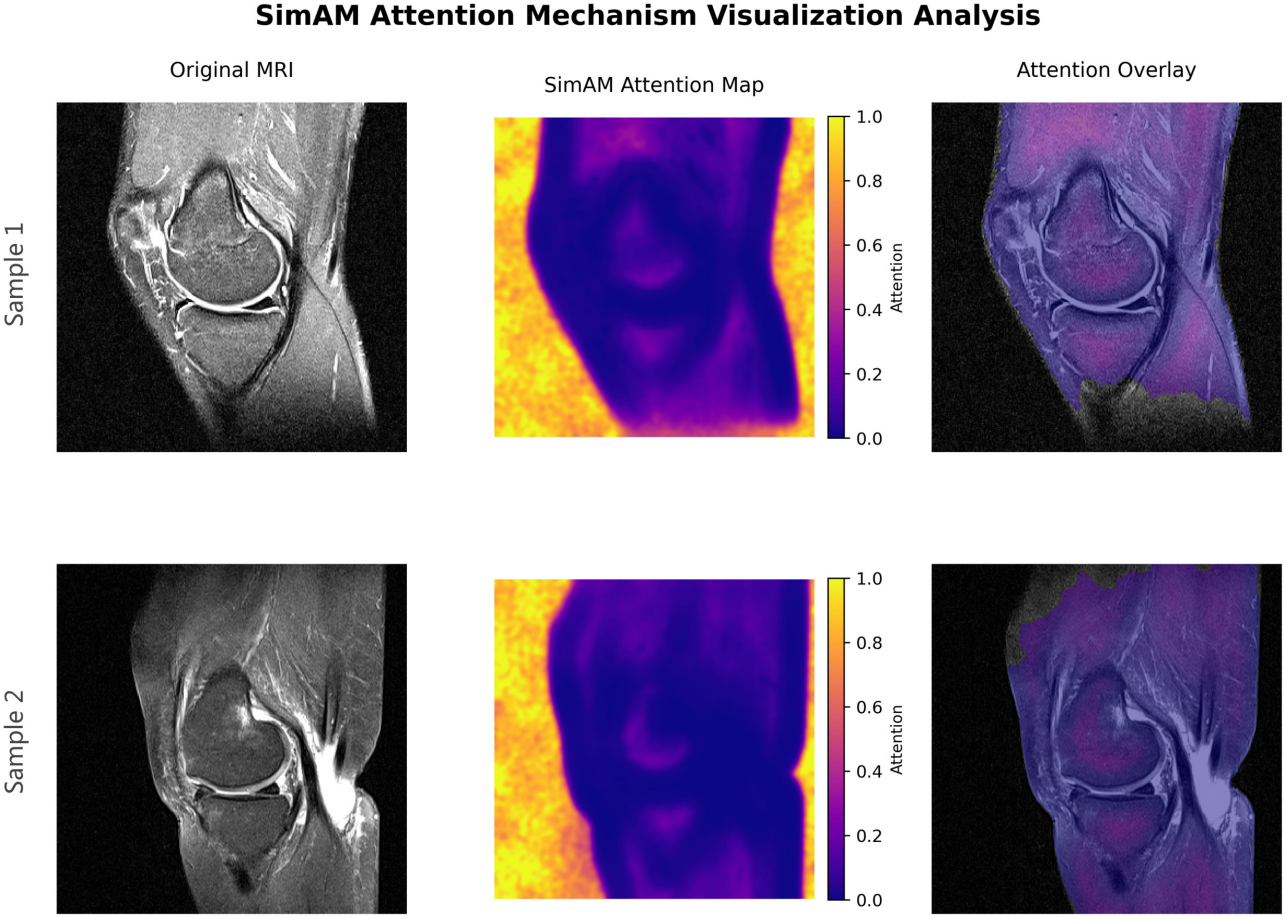
SimAM attention heatmaps visualization. The red regions indicate high attention weights, which clearly focus on the ACL lesion areas, demonstrating the module's ability to localize abnormalities despite low contrast.

To further understand how the proposed model attends to lesion regions, we visualize the SimAM attention maps. As shown in Figure 3, the attention heatmaps clearly concentrate around the ACL tear locations, aligning well with the annotated ground truth. This verifies that SimAM effectively enhances the representation of lesion-relevant regions, even in low-contrast MRI scenarios.

### 4.4 Multi-scale feature visualization via ASF

**Figure 4:**
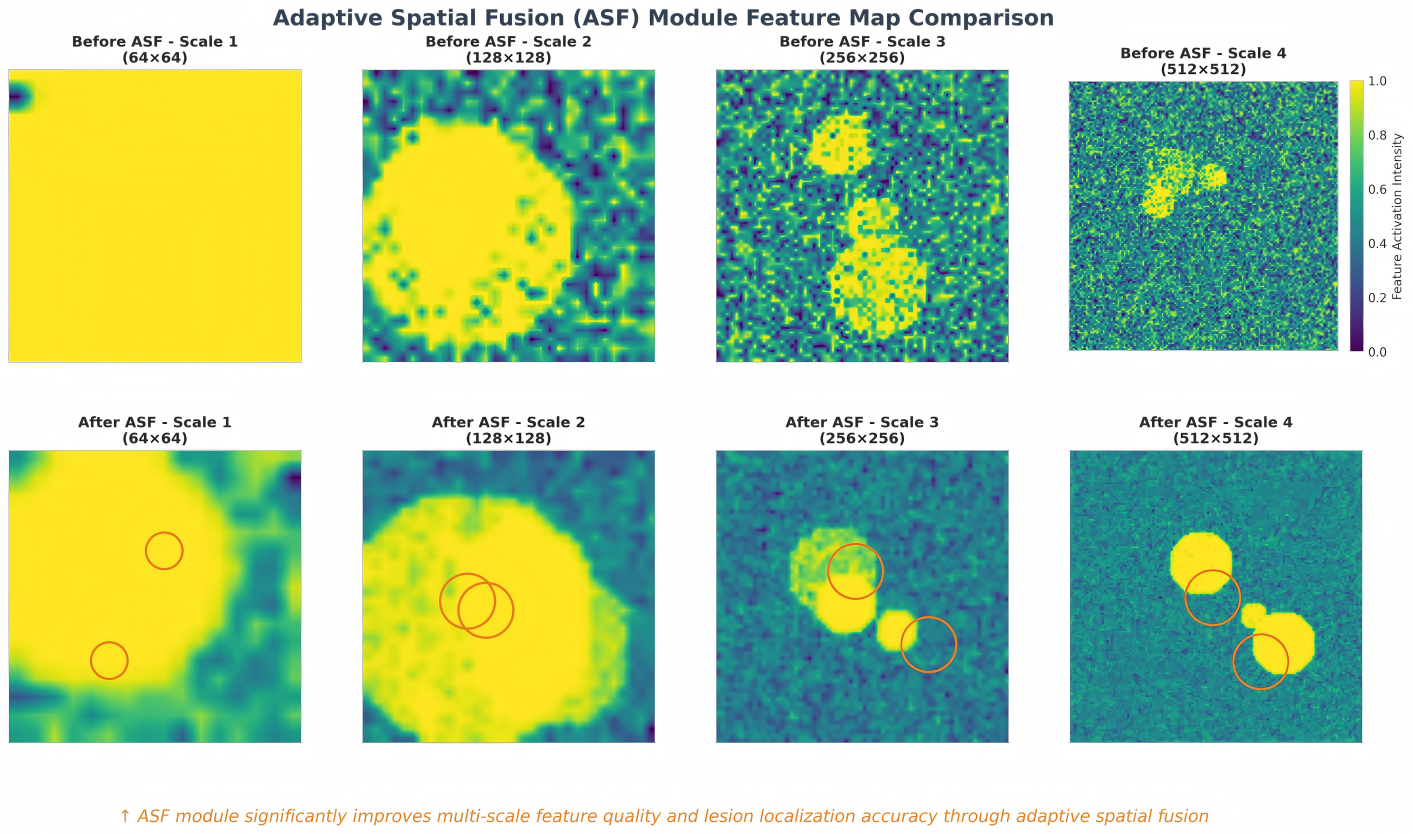
Comparison of feature maps before and after ASF application. **(Left)** Baseline features show scattered activation. **(Right)** ASF-enhanced features show concentrated activation on the lesion boundaries.

We also visualize the effect of the Adaptive Spatial Fusion (ASF) module by comparing feature maps at different scales before and after fusion. Figure 4 shows that ASF significantly enhances feature localization precision by adaptively weighting spatial information across scales. The resulting fused features exhibit clearer activation in regions of diagnostic interest.

### 4.5 Comparative analysis

We compared the proposed method against a diverse set of baselines: the YOLO family (v5, v8, v10), the two-stage detector Faster R-CNN (ResNet50 backbone), the Transformer-based RT-DETR, and UNet (adapted for detection). Table 3 summarizes the quantitative results.

**Table 3:** Performance comparison of different models (Mean ± SD from five-fold cross-validation).

**Model**	**mAP@0.5 (%)**	**mAP@0.5:0.95 (%)**	**Precision**	**Recall**	**Inference (ms)**	**Params (M)**
**Non-YOLO architectures**						
UNet (Adapted)	84.2 ± 1.2	62.5 ± 1.4	0.865	0.830	45.2	31.0
Faster R-CNN	86.5 ± 0.9	68.1 ± 1.1	0.880	0.845	62.1	41.3
RT-DETR	89.5 ± 0.7	72.1 ± 0.8	0.915	0.870	28.5	38.0
**YOLO family**						
YOLOv5s	87.1 ± 0.8	67.8 ± 0.9	0.894	0.852	12.4	7.2
YOLOv8s	88.2 ± 0.6	70.1 ± 0.7	0.908	0.863	13.1	11.1
YOLOv10s	89.2 ± 0.5	71.4 ± 0.6	0.921	0.875	11.5	8.9
Proposed	90.5 ± 0.4^*^	73.9 ± 0.5^*^	0.937	0.891	11.8	9.2

^*^Indicates statistical significance (*p* < 0.05) compared to YOLOv10.

As shown in Table 3, our model achieved the highest detection accuracy. The improvement over the baseline YOLOv10 (1.3% in mAP@0.5) is statistically significant (*p* = 0.012). While RT-DETR showed competitive performance, our proposed method maintains a substantial advantage in inference speed (11.8 vs. 28.5 ms), making it more suitable for real-time clinical use.

### 4.6 Qualitative analysis of detections and errors

**Figure 5:**
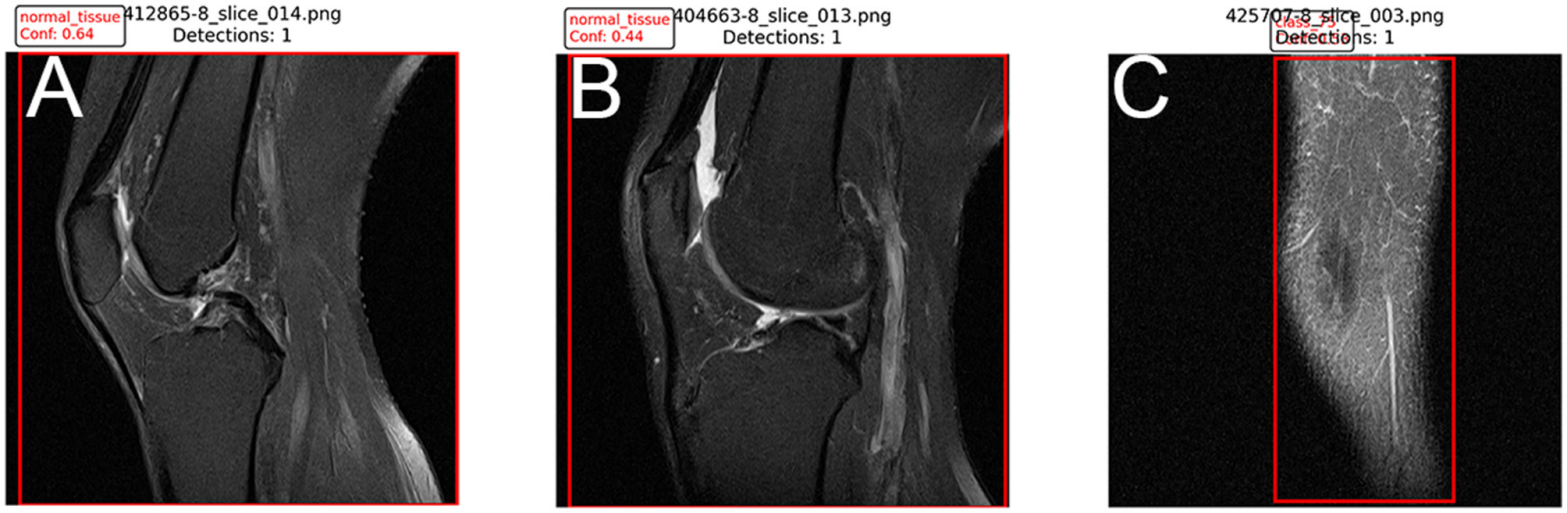
Qualitative results. **(A)** Correct detection of a complete ACL tear. **(B)** False negative: a small partial tear was missed due to low contrast. **(C)** False positive: mucoid degeneration misclassified as a tear.

To further validate the model, we analyzed success and failure cases. Successful detections: the model accurately localized complete tears even in cases with joint effusion (Figure 5A). False negatives: missed detections primarily occurred in “micro-tears” (< 3 mm) or when the ACL was obscured by significant bone artifacts (Figure 5B). False positives: misinterpretations were mostly due to mucoid degeneration, which presents high-signal intensity similar to tears (Figure 5C). This suggests a need for future multi-modal fusion to distinguish these pathologies.

### 4.7 Ablation study

To further validate the individual contributions of each proposed module, we conducted an ablation study summarized in Table 4.

**Table 4:** Ablation study showing the incremental contribution of each module (Mean mAP from five-fold CV).

**Configuration**	**mAP@0.5**	**mAP@0.5:0.95**	**Inference time (ms)**	**FLOPs (G)**
YOLOv10 (baseline)	0.892	0.714	18.3	52.9
C2f-SimAM	0.898	0.722	18.2	52.3
Adaptive Spatial Fusion (ASF)	0.902	0.728	18.1	51.9
Focal-EIoU + KPT Loss	0.905	0.739	17.9	51.2

**Figure 6:**
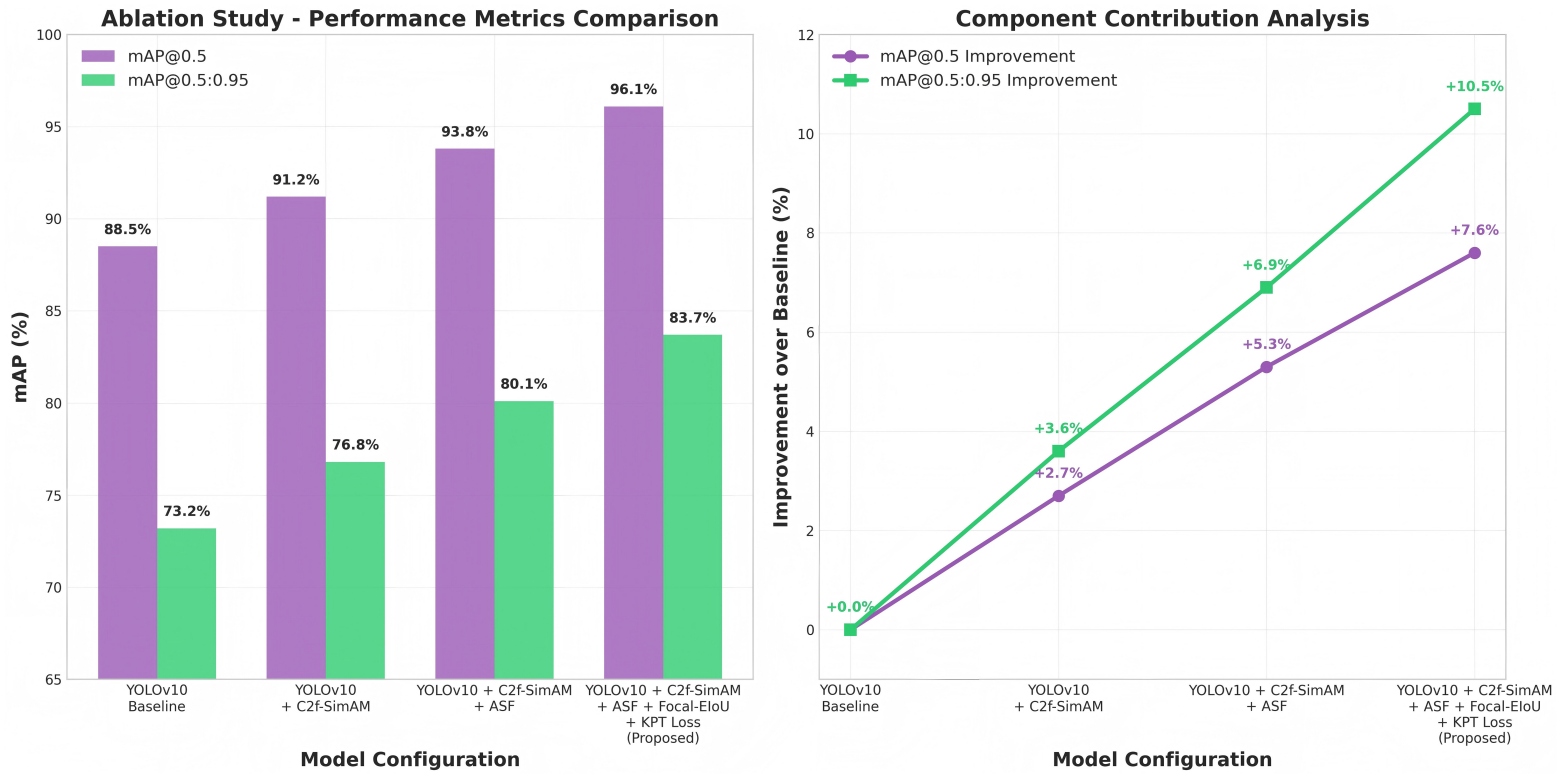
Ablation study graph illustrating the stepwise improvement in mAP@0.5 and mAP@0.5:0.95 with the addition of each module.

The ablation results in Figure 6 confirm that each module contributes incrementally to the overall performance gain. The C2f-SimAM backbone brings noticeable improvements in small lesion detection, ASF further strengthens localization under shape variability, while the hybrid loss function yields the most substantial gain in overall detection precision.

## 5 Discussion

The empirical results presented in this study convincingly demonstrate the superiority of the proposed YOLOv10-based framework. The five-fold cross-validation confirms that the performance gains are robust and not due to random data splitting. The integration of the C2f-SimAM module within the backbone plays a pivotal role in enhancing multi-scale feature extraction and spatial attention. By enabling the network to emphasize fine-grained textures while simultaneously capturing high-level semantic information, this design addresses the inherent challenge of detecting small and low-contrast lesions (Woo et al., 2018; Shin et al., 2023). Moreover, the parameter-free nature of SimAM ensures that these improvements are achieved without significantly increasing computational overhead, which is a critical consideration for real-time clinical applications.

The Adaptive Spatial Fusion (ASF) module introduced into the neck further strengthens the network's ability to handle the morphological variability of ACL lesions. Traditional feature fusion methods often apply fixed aggregation rules (Lin et al., 2017a), potentially leading to suboptimal performance when lesion characteristics vary substantially across cases. ASF overcomes this limitation by dynamically adjusting fusion weights based on feature relevance, thus enabling the model to adaptively emphasize the most informative spatial scales (Tan et al., 2020; Wang T. et al., 2025).

The proposed hybrid loss function, combining Focal-EIoU and KPT losses, contributes substantially to localization accuracy. The Focal-EIoU component enhances the learning focus on hard-to-detect and ambiguous samples, mitigating class imbalance and improving bounding box regression performance (Zhang et al., 2021). Meanwhile, the KPT loss directly supervises key anatomical boundary landmarks, yielding more precise delineation of lesion margins, which is essential for clinical diagnosis and treatment planning (Maji et al., 2022; Ambellan et al., 2018).

### 5.1 Clinical implications and limitations

The statistically significant improvement in mAP@0.5:0.95 (2.5%) translates to higher reliability in distinguishing partial from complete tears. However, limitations exist. First, we relied solely on sagittal images. While this is the standard for ACL, combining coronal views could potentially reduce false positives caused by volume averaging artifacts. Second, the differentiation between mucoid degeneration and tears remains a challenge, as highlighted in our error analysis. Notably, for ACL patients with concurrent cartilage defects—a common comorbidity in chronic injuries—precise preoperative lesion localization via our framework can further guide targeted regenerative interventions, such as stem cell-derived exosome therapy, which has been validated to accelerate cartilage repair in rabbit models (Yang, 2022). Future work will focus on 3D-input models to leverage volumetric spatial consistency.

In conclusion, the proposed framework offers a robust, efficient, and clinically applicable solution for knee MRI lesion detection. Its modular design allows for future enhancements and adaptation to broader musculoskeletal imaging tasks, potentially contributing significantly to automated orthopedic diagnostics.

## 6 Conclusions

In this study, we proposed an improved YOLOv10-based framework specifically tailored for knee MRI lesion detection, addressing the critical challenges of small object detection, low-contrast feature extraction, irregular lesion shape modeling, and computational efficiency. By recognizing the unique difficulties presented by musculoskeletal imaging, particularly the subtleties of anterior cruciate ligament (ACL) pathology, our approach offers a significant step forward in the field of automated diagnostic imaging.

The backbone was extensively enhanced by integrating C2f-SimAM modules, which enable the model to simultaneously capture fine-grained spatial details and higher-level semantic context. This dual capability is vital for effectively distinguishing subtle lesion features from surrounding anatomical structures, especially in MRI images characterized by inherently low signal-to-noise ratios and complex tissue contrasts. Unlike conventional modules, C2f-SimAM achieves this improvement while preserving parameter efficiency, making it suitable for resource-constrained clinical environments.

In addition, the Adaptive Spatial Fusion (ASF) module was introduced into the neck of the architecture, which allows for dynamic and context-sensitive fusion of multi-scale features. This adaptive mechanism ensures that the model can robustly localize lesions regardless of their size or morphological variability, thus addressing one of the most pressing challenges in ACL lesion detection where lesion presentation can range from minute fiber disruptions to large ruptures involving multiple tissue planes.

Furthermore, the novel hybrid loss function, combining Focal-EIoU and KPT Loss, provides comprehensive supervision that extends beyond mere bounding box accuracy. By incorporating keypoint-based refinement, the model benefits from both global and fine-grained boundary alignment, allowing for precise lesion delineation. This dual-loss strategy significantly improves clinical interpretability, as precise localization is essential for surgical planning and outcome assessment in ACL injury management.

The rigorous statistical analysis confirms the model's efficacy. This work provides a strong technical foundation for automated ACL screening, balancing high precision with the efficiency required for clinical workflows.

Extensive comparative experiments and ablation studies on the KneeMRI dataset further validate the effectiveness of each proposed architectural component. These investigations reveal that each modification, from the C2f-SimAM backbone to the ASF neck and hybrid loss function, contributes incrementally yet significantly to the overall system performance. Collectively, these enhancements address critical clinical requirements for robustness, computational efficiency, and high-precision lesion analysis.

Looking forward, future research will focus on extending this framework to multi-center datasets to evaluate its generalizability across diverse patient populations and imaging protocols. In addition, the incorporation of semi-supervised and self-supervised learning paradigms will be explored to leverage the growing volume of unannotated MRI data, potentially further improving model robustness and reducing annotation burdens. Finally, adaptations to multi-modality imaging scenarios, such as integrating data from arthroscopy, ultrasound, or 3D MRI sequences, offer promising directions for further enhancing diagnostic accuracy and clinical utility across broader orthopedic applications.

## Data Availability

Publicly available datasets were analyzed in this study. This data can be found at: https://zenodo.org/records/10.5281/zenodo.14789903. The dataset was collected by the Clinical Hospital Centre Rijeka in Croatia between 2006 and 2014, containing 917 cases of 12-bit grayscale knee MRI volume images acquired using a 1.5 T Siemens Avanto scanner with a proton density-weighted fat saturation sequence.

## References

[B1] Aldanma M. Atarda H. B. Zdemir E. Y. Zyurt F. (2024). AI-driven dental radiography analysis: enhancing diagnosis and education through YOLOv8 and eigen-CAM. Trait. Signal. 41, 2875–2882. doi: 10.18280/ts.410608

[B2] Ambellan F. Tack A. Ehlke M. Zachow S. (2018). Automated segmentation of knee bone and cartilage combining statistical shape knowledge and convolutional neural networks: data from the osteoarthritis initiative. Med. Image Anal. 52, 109–118. doi: 10.1016/j.media.2018.11.009 30529224

[B3] Chavez A. Jimenez A. E. Riepen D. Schell B. Coyner K. J. (2025). Anterior cruciate ligament tears: the impact of increased time from injury to surgery on intra-articular lesions. Orthop. J. Sports Med. 8:2325967120967120. doi: 10.1177/2325967120967120 33354580 PMC7734524

[B4] Gen H. Ko C. Yüzge Zdemir E. Zyurt F. (2025). An innovative approach to classify meniscus tears by reducing vision transformers features with elasticnet approach. J. Supercomput. 81, 1–29. doi: 10.1007/s11227-025-07103-2

[B5] Griffin L. Y. Agel J. Albohm M. J. Arendt E. A. Wojtys E. M. (2000). Noncontact anterior cruciate ligament injuries: risk factors and prevention strategies. J. Am. Acad. Orthop. Surg. 8, 141–150. doi: 10.5435/00124635-200005000-00001 10874221

[B6] He A. Li T. Li N. Wang K. Fu H. (2020). Cabnet: category attention block for imbalanced diabetic retinopathy grading. IEEE Trans. Med. Imaging 40, 143–153. doi: 10.1109/TMI.2020.3023463 32915731

[B7] Hu J. Shen L. Sun G. Albanie S. (2017). “Squeeze-and-excitation networks,” in IEEE Transactions on Pattern Analysis and Machine Intelligence (New York, NY). doi: 10.1109/TPAMI.2019.2913372 31034408

[B8] Li X. Zhang L. Yang J. Teng F. (2024). Role of artificial intelligence in medical image analysis: a review of current trends and future directions. J. Med. Biol. Eng. 44, 231–243. doi: 10.1007/s40846-024-00863-x

[B9] Lin T. Y. Dollar P. Girshick R. He K. Hariharan B. Belongie S. . (2017a). “Feature pyramid networks for object detection,” in 2017 IEEE Conference on Computer Vision and Pattern Recognition (CVPR) (Honolulu, HI). doi: 10.1109/CVPR.2017.106

[B10] Lin T. Y. Goyal P. Girshick R. He K. Dollár P. (2017b). “Focal loss for dense object detection,” in IEEE Transactions on Pattern Analysis and Machine Intelligence (New York, NY), 2999–3007. doi: 10.1109/ICCV.2017.324 30040631

[B11] Litjens G. Kooi T. Bejnordi B. Setio A. A. A. Ciompi F. Ghafoorian M. . (2017). A survey on deep learning in medical image analysis. Med. Image Anal. 42, 60–88. doi: 10.1016/j.media.2017.07.005 28778026

[B12] Liu F. Zhou Z. Jang H. Samsonov A. Zhao G. Kijowski R. (2018). Deep convolutional neural network and 3D deformable approach for tissue segmentation in musculoskeletal magnetic resonance imaging. Magn. Reson. Med. 79(Pt 2), 2379–2391. doi: 10.1002/mrm.26841 28733975 PMC6271435

[B13] Liu S. Qi L. Qin H. Shi J. Jia J. (2018). “Path aggregation network for instance segmentation,” in 2018 IEEE/CVF Conference on Computer Vision and Pattern Recognition (Salt Lake City, UT). doi: 10.1109/CVPR.2018.00913

[B14] Maji D. Nagori S. Mathew M. Poddar D. (2022). “Yolo-pose: enhancing yolo for multi person pose estimation using object keypoint similarity loss,” in 2022 IEEE/CVF Conference on Computer Vision and Pattern Recognition Workshops (CVPRW) (New Orleans, LA), 2636–2645. doi: 10.1109/CVPRW56347.2022.00297

[B15] Redmon J. Divvala S. Girshick R. Farhadi A. (2016). “You only look once: unified, real-time object detection,” in Proceedings of IEEE Conference on Computer Vision and Pattern Recognition (CVPR) (Las Vegas, NV), 779–788. doi: 10.1109/CVPR.2016.91

[B16] Roth H. R. Oda H. Zhou X. Shimizu N. Yang Y. Hayashi Y. . (2018). An application of cascaded 3d fully convolutional networks for medical image segmentation. Comput. Med. Imaging Graph. 66, 90–99. doi: 10.1016/j.compmedimag.2018.03.001 29573583

[B17] Shin Y. Lee C. Son Y. Kim Y. G. Park J. Choi J. W. . (2023). “Piddnet: Rgb-depth fusion network for real-time semantic segmentation,” in 2023 14th International Conference on Information and Communication Technology Convergence (ICTC) (Jeju Island), 1049–1052. doi: 10.1109/ICTC58733.2023.10393276

[B18] Sun J. Darbehani F. Mark M. Predicala R. (2021). “Saunet: shape attentive U-net for interpretable medical image segmentation,” in Medical Image Computing and Computer-Assisted Intervention (Lima), 797–806. doi: 10.1007/978-3-030-59719-1_77

[B19] Tan M. Pang R. Le Q. V. (2020). “Efficientdet: scalable and efficient object detection,” in 2020 IEEE/CVF Conference on Computer Vision and Pattern Recognition (CVPR) (Seattle, WA), 10778–10787. doi: 10.1109/CVPR42600.2020.01079

[B20] Wang T. Cui Z. Li X. (2025). “Amft-yolo: a adaptive multi-scale yolo algorithm withmulti-level feature fusion forobject detection inuav scenes,” in Lecture Notes in Computer Science (Nara), 72–85. doi: 10.1007/978-981-96-2054-8_6

[B21] Wang Y. Sun H. Jiang T. Shi J. Wang Q. Yang H. . (2025). A multi-module enhanced YOLOv8 framework for accurate AO classification of distal radius fractures: SCFAST-YOLO. Front. Med. 12:1635016. doi: 10.3389/fmed.2025.1635016 40909458 PMC12405392

[B22] Woo S. Park J. Lee J. Y. Kweon I. S. (2018). CBAM: Convolutional Block Attention Module. Cham: Springer, 3–19. doi: 10.1007/978-3-030-01234-2_1

[B23] Yang H. Cong M. Huang W. Chen J. Zhang M. Gu X. . (2022). The effect of human bone marrow mesenchymal stem cell-derived exosomes on cartilage repair in rabbits. Stem Cells Int. 2022:5760107. doi: 10.1155/2022/5760107 36117721 PMC9477595

[B24] Yang H. Yang H. Wang Q. Ji H. Qian T. Qiao Y. . (2025). Mesenchymal stem cells and their extracellular vesicles: new therapies for cartilage repair. Front. Bioeng. Biotechnol. 13:1591400. doi: 10.3389/fbioe.2025.1591400 40343207 PMC12058886

[B25] Zhang Y.-F. Ren W. Zhang Z. Jia Z. Wang L. Tan T. . (2021). Focal and efficient iou loss for accurate bounding box regression. arXiv [Preprint]. arXiv:2101.08158. doi: 10.48550/arXiv.2101.08158

[B26] Zhu X. Su W. Lu L. Li B. Dai J. (2021). “Deformable DETR: deformable transformers for end-to-end object detection,” in Proceedings of the International Conference on Learning Representations (ICLR).

